# Isolation, identification, and antimicrobial effect analysis of active components from *Humulus scandens* against *Phytophthora nicotianae*

**DOI:** 10.3389/fpls.2026.1753587

**Published:** 2026-03-05

**Authors:** Xiaoyun Wu, Yuxuan Liu, Deqiang Qin, Dongmei Liu, Yongsheng Ren, Jin Tian, Siyue Lan, Xiao Ding, Xiaoping Qin

**Affiliations:** 1 College of Plant Protection, Yunnan Agricultural University, Kunming, China; 2 Leaf Tobacco Production Technology Center, Yunnan Tobacco Company, Qujing, China; 3 State Key Laboratory of Phytochemistry and Plant Resources in West China, Kunming Institute of Botany, Chinese Academy of Sciences, Kunming, China

**Keywords:** antimicrobial activity, chemical structure, *Humulus scandens*, isolation, *Phytophthora nicotianae*

## Abstract

Monomeric compounds from *Humulus scandens* that effectively inhibit *Phytophthora nicotianae* were isolated, and their antimicrobial effects were analyzed. Methanol extracts were isolated using a combination of activity tracking and chemical separation methods. Compound structures were identified using NMR and other techniques. Antimicrobial activity against *P. nicotianae* was assessed via the mycelial growth rate method with mycelial morphology further observed using optical microscopy and scanning electron microscopy. Five compounds were isolated from the ethyl acetate (EtOAc) layer of *H. scandens*, namely, chromone (compound 1), tectochrysin (compound 2), isorhamnetin (compound 3), hyperoside (compound 4), and Apigenin 7-glucoside (compound 5). All compounds exhibited varying degrees of antimicrobial activity. Compounds 1 and 5 demonstrated superior inhibitory effects, with EC_50_ values of 51.70 and 31.71 μg/ml and MIC values of 400 and 200 μg/mL, respectively. Microscopic examination revealed that compounds 1 and 5 induced distortion, deformation, shrinkage, collapse, and damage in *P. nicotianae* mycelia. Additionally, they increased membrane permeability and inhibited mycelial growth by disrupting cellular integrity. This study provides lead compounds for developing green botanical pesticides against tobacco black shank disease and offers data to support green agriculture initiatives.

## 1 Introduction

*Phytophthora nicotianae* is a plant pathogenic oomycete within the order Peronosporales (Cochran et al., 2024). It exhibits strong pathogenicity and adaptability, proliferating rapidly under high-temperature and high-humidity conditions. The resultant tobacco black shank disease is a devastating soil-borne ailment (Fang et al., 2016), causing significant yield loss and economic damage. Current management strategies include agricultural, biological, and chemical controls. However, as tobacco black shank is soil-borne, agricultural control alone is ineffective (Antonopoulos et al., 2010). Biological control, while environmentally sustainable, acts slowly and is highly environment-dependent (Ding et al., 2021), limiting its widespread adoption. Chemical control remains the primary method (Jing et al., 2017) but faces limitations due to resistance, residue, and resurgence (3R problems). Thus, identifying novel control strategies is imperative.

Botanical pesticides are natural pesticides usually extracted/derived from plants (Sengül Demirak and Canpolat, 2022), containing bioactive ingredients such as fungicides, insecticides, and herbicides. These represent promising green alternatives (Wang, 2022). Key constituents include flavonoids, terpenoids, alkaloids, and essential oils (Schnarr et al., 2022; Chaudhari et al., 2021). For example, flavonoids from licorice exhibit inhibitory effects against *Fusarium oxysporum* (Duan et al., 2025); abietane diterpenoids from *Salvia canariensis* suppress plant pathogenic fungi (López-Cabeza et al., 2024); alkaloid derivatives inhibit *Botryosphaeria theobromae* (Zhang et al., 2025); and *Pelargonium graveolens* essential oil targets *Agrotis ypsilon* larvae (Moustafa et al., 2021). Eudesmanolides and guaianolides display herbicidal potential (Zorrilla et al., 2024). Recently, botanical pesticides have been widely studied for their low toxicity, low residue, environmental compatibility, reduced resistance development, and friendliness to non-target organisms. Isolating bioactive monomers from natural sources remains a focus in chemistry, pharmacology, and pesticide research (Yang et al., 2022; Prastiwi et al., 2023; Saravanan, 2022).

*Humulus scandens*, also known as Lalayang or Wuzhualong, a perennial climbing herb of Moraceae in Urticales, is a fast-propagating invasive weed (Liu et al., 2025b). *Humulus scandens* can produce secondary metabolites including flavonoids, sterols, terpenoids, alkaloids, volatile oils, and lignins during growth (Carbone and Gervasi, 2022; Wang et al., 2025), with significant bioactivity. Its n-hexane extract has acaricidal activity (Tao et al., 2011); volatiles such as myrcene, benzaldehyde, and linalool attract the Asian corn borer to oviposit (Zhang et al., 2018). The volatile substances released from the stems and leaves of *H. scandens* had a significant inhibitory effect on the growth of the seedlings of the four tested plants (Liu et al., 2011). The extracts of *H. scandens* roots obtained by three chemical solvents can destroy the leaf membrane system of *Alternanthera philoxeroides* and inhibit plant growth (Wang et al., 2021). These studies provide a theoretical basis for the development of herbicides. In addition, *H. scandens* also has rich pharmacological activities, including antibacterial (Carbone and Gervasi, 2022), anti-inflammatory (Wu and Su, 2010; Liu et al., 2023), antidiarrheal (Hao et al., 2024; Lv and Feng, 2013), immunosuppressive (Feng et al., 2014), antioxidation (Dang et al., 2023), antitumor (Carbone and Gervasi, 2022), and other functions.

Previous studies on *H. scandens* have focused on its secondary metabolites (Carbone and Gervasi, 2022) and crude extract bioactivity (Wang et al., 2024), but few have identified its active constituents against specific agricultural pathogens. This work addresses this critical gap by demonstrating the potent inhibitory activity of ethyl acetate (EtOAc) extract against *P. nicotianae*. Therein lies our primary novelty; we isolate and characterize the specific bioactive compounds within this extract responsible for anti-oomycete activity, a substantial advancement beyond the existing paradigm where only crude extract activity against pathogens has been reported and the precise bioactive monomers remain unidentified. Furthermore, current management of *P. nicotianae* relies heavily on chemical controls prone to resistance development, while biological alternatives often face challenges in formulation stability and storage (Zhou et al., 2025). Consequently, this study uniquely addresses the research gap for novel, sustainable control agents by providing foundational evidence that *H. scandens* constituents offer a viable template for developing botanical fungicides. The isolation of active monomers presented here directly facilitates their potential formulation into cost-effective, environmentally compatible, and readily deployable green pesticides for managing tobacco black shank disease.

## 2 Materials and methods

### 2.1 Materials

Aerial parts of *H. scandens* were collected in Anyang, Henan, China (35°12′N, 113°37′E) in July 2022. Specimens were authenticated by the Kunming Institute of Botany, Chinese Academy of Sciences (Number 2022017).

*Phytophthora nicotianae*, *Bipolaris maydis*, *Alternaria alternata*, *Corynespora cassiicola*, *Fusarium oxysporum*, *Magnaporthe oryzae*, *Alternaria panax*, *Colletotrichum fructicola*, *Mycocentrospora acerina*, and *Alternaria solani* used in the experiment were provided by the Laboratory of Biological Pesticide Development and Utilization, Yunnan Agricultural University (Yang et al., 2025).

### 2.2 Apparatus

The following were utilized in the experiment: normal-phase silica gel (300–400, 200–300, 60–80 mesh) and TLC plates (Qingdao Ocean Chemical Co., Ltd., Shandong, China), RP-18 silica gel (40–63 μM; Merck, Darmstadt, Germany), MCI gel (75–150 μM; Chengdu Scientific Biochemistry Co., Ltd., Sichuan, China), Sephadex LH-20 (40–70 μM; Pharmacia, USA. Sweden, USA), YMC-Pack ODS-A column (5 μM, 10 × 250 mm; Shenzhen Kemis Technology Co., Ltd., Guangzhou, China), HPLC-grade acetonitrile and methanol (Tianjin Kemiou Co., Tianjin, China), distilled water (Wahaha Group, Hangzhou, China), 96-well plates (Corning, Corning, USA), rotary evaporator (Heidolph, Germany), preparative HPLC (Jiangsu Hanbang Technology Co., Ltd., Huaian, China), climate chamber (Ningbo Southeast Instrument Co., Ltd., Zhejiang, China), autoclave (Zealway, Delaware, USA), and clean bench (Sujing Group Antai Co., Suzhou, China).

### 2.3 Extraction of *Humulus scandens*

The collected 20 kg of fresh *H. scandens* was shade-dried and crushed. Then, it was extracted three times with methanol at room temperature. The extracts were combined and concentrated under reduced pressure to yield 5 L of crude methanol extract. Next, the crude extract was completely dissolved in pure water and again extracted using the same amount of petroleum ether, ethyl acetate, and n-butanol, and the cycle was repeated three times to obtain 372 g of EtOAc layer.

### 2.4 Compound separation and identification

The EtOAc layer of *H. scandens* was subjected to silica gel column chromatography using a silica gel column (10 s) and eluted with PE-EA (50:1-pure EA) to obtain 12 fractions (Lx.1–Lx.12).

Lx.5 (30.6 g) was first purified by MCI reverse column chromatography (10% MeOH-H_2_O-pure MeOH-H_2_O) to obtain eight fractions (Lx.5.A–Lx.5.H) and then repeated by normal phase silica gel column chromatography (PE-EA or PE-ACE mixed solution elution) and gel column chromatography (MeOH-H_2_O or MeOH-DCM 6:4). Finally, compounds 1 (247 mg), 2 (63 mg), 3 (77 mg), 4 (62 mg), and 5 (77 mg) were obtained (purities ≥ 95%).

The structures of all compounds were determined by measuring the 1H, 13C, DEPT nuclear magnetic resonance spectroscopy data, and mass spectrometry data, combined with SciFinder search and literature review for data comparison.

### 2.5 Antimicrobial activity screening of crude extract

The antifungal activity of the EtOAc layer of *H. scandens* against 10 pathogens was screened using the mycelium growth rate method (Lin et al., 2022). Initially, 10 kinds of pathogenic bacteria were activated. Under sterile conditions, 33.75 mL of liquid medicine was added to 566.25 mL of potato dextrose agar (PDA), and a drug-containing medium with a concentration of 3 mg/mL was prepared. After high-temperature sterilization, the inverted plate operation was carried out on the ultra-clean bench to make a drug-containing plate. Finally, a tested bacterium with a diameter of 5 mm was inoculated in the center of the drug-containing plate. Sterile water was set as the blank control, and sterile water containing 0.5% methanol was set as the negative control. Each treatment was repeated three times. After 72 h of dark inverted culture in a constant temperature incubator at 27 °C ± 2°C and 85% ± 5% RH, the colony diameter was measured by the cross-crossing method, and the inhibition rate was calculated as follows:


$$\text{Inhibition }\left(\%\right)\;=\;({\text{OD}}_{\text{CK}}-\text{ OD Compound}/{\text{OD}}_{\text{CK}}-\text{OD Fungus Cake})\;\times \;100\%$$

$$\left(\text{Note}:{\text{ OD}}_{\text{CK}}=\text{ OD }-\text{ control}\right)$$

### 2.6 Antimicrobial activity screening of compounds and EC_50_ determination

According to the method of Section 2.5, the compounds were prepared into a drug-containing plate with a concentration of 100 μg/mL to screen *P. nicotianae.* When the compound showed good activity against the tested bacteria, then the medium was prepared with five concentration gradients (100, 50, 25, 12.5, and 6.25 μg/mL) to determine the EC_50_. The other operations were the same as above.

### 2.7 The MIC of the compounds against *Phytophthora nicotianae*

The MIC of the compounds against *P. nicotianae* was determined by the 96-well plate broth dilution method (Silva et al., 2025). The mother liquor was prepared and diluted into different concentrations (12.5, 25, 50, 100, 200, 400, 800 μg/mL). *Phytophthora nicotianae* was treated with 0.1% KNO_3_ to induce sporulation, and a spore suspension with a concentration of 1 × 10^6^ CFU/mL was prepared for use. Under sterile conditions, the drug-containing medium and spore suspension volume were injected into a 96-well plate, and a control was set, with three replicates for each treatment. Cultured in an incubator at 28 °C, when the control medium showed obvious mycelial growth, the treatment did not have any minimum drug concentration for mycelial growth, which was determined as the MIC.

### 2.8 Effect of compounds on mycelial morphology of *Phytophthora nicotianae*

The effect of monomeric compounds on *P. nicotianae* was observed by the coating plate method under a microscope. The spore suspension was evenly coated on the medium plate with EC_50_ concentration, and the control was set up. The spore suspension was placed in the incubator at 28°C, and the morphological changes of the mycelium were observed under light microscopy.

Further observation was carried out by scanning electron microscopy (SEM). When the mycelium of the control group was full, the medium of different treatments was cut into small pieces, fixed to the sample table, and frozen in supercooled liquid nitrogen for 2 min. Then, the sample was transferred into the preparation chamber, and the cold table was sublimated at −140°C and −100 °C for 15 min. The samples were coated twice for 60 s each and then placed under a frozen scanning electron microscope to observe the microscopic morphological characteristics of the mycelium.

### 2.9 Effects of compounds on the cell membrane integrity of *Phytophthora nicotianae*

Propidium iodide (PI) cannot penetrate the intact living cell membrane but can enter cells through damaged cells and combine with DNA to produce fluorescence. Using the fluorescence labeling method, the drug-treated mycelia were washed twice with PBS buffer and transferred to the slide. Then, 50 μL of PI staining solution (1 g/L) was added dropwise to the slide and stained in the dark for 15 min, and the excess dye was removed by washing with PBS buffer. Finally, the glass was covered, and the inverted fluorescence microscope was used to observe the parameters of the excitation wavelength of 535 nm and the emission wavelength of 617 nm (Liu et al., 2025a).

### 2.10 Statistical analysis

In order to ensure the reliability and repeatability of the results, each experiment was repeated three times, and the data were expressed as mean ± standard deviation (SD). The experimental data were analyzed using Excel 2010, and then IBM SPSS Statistics 25 was used for comprehensive statistical analysis. One-way analysis of variance (ANOVA) combined with the Tukey test was used for significance analysis (*p* < 0.05). Graphs were generated using Origin 2022 (version 9.900220; OriginLab, Northampton, MA, USA).

## 3 Results

### 3.1 Activity screening

**Figure 1:**
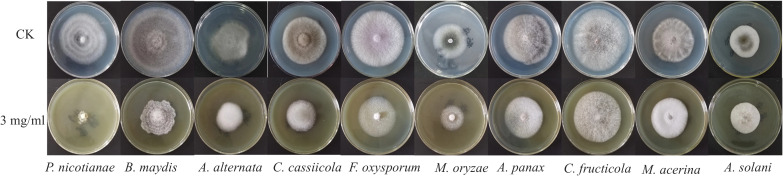
Screening of antimicrobial activity of the EtOAc layer extract from *Humulus scandens*.

The antimicrobial activity of the EtOAc layer of *H. scandens* against 10 pathogenic bacteria is shown in **Figure 1** and **Table 1**. At the concentration of 3 mg/mL, the crude extract had good inhibitory activity against *P. nicotianae*, and the inhibition rate reached 82.81%. It has certain activity against other pathogens, but the effect is not obvious, and the inhibition rate is below 50%. Consequently, *P. nicotianae* was selected for activity-guided compound isolation.

**Table 1:** Screening of antimicrobial activity of *Humulus scandens* EtOAc extract.

Phytopathogen	Inhibition rate (%; mean ± SD)	Phytopathogen	Inhibition rate (%; mean ± SD)
*P. nicotianae*	82.81 ± 0.47a	*M. oryzae*	28.68 ± 0.54e
*B. maydis*	46.40 ± 1.60b	*A. panax*	23.48 ± 0.54f
*A. alternata*	40.16 ± 0.34c	*C. fructicola*	19.2 ± 0.32g
*C. cassiicola*	35.28 ± 0.32d	*M. acerina*	18.11 ± 0.45g
*F. oxysporum*	30.42 ± 0.46e	*A. solani*	9.73 ± 0.023h

The data were expressed as mean ± standard deviation (*N* = 3), and the Tukey test was used to test the significance of the difference. Different lowercase letters in the table indicate that the *p* < 0.05 level was significantly different (the same applies to **Table 2**).

The activity tracking revealed the activity of 12 fractions (Lx.1–Lx.12) obtained from the crude extract fraction, as shown in **Table 2**. At the concentration of 1 mg/mL, all fractions except Lx.1 had activity against *P. nicotianae*. Lx.5 (65.74%) had better activity, so it was selected for further separation.

**Table 2:** Activity of various fractions from the EtOAc layer of *Humulus scandens* against *Phytophthora nicotianae*.

Fractions	Inhibition rate (%; mean ± SD)	Fractions	Inhibition rate (%; mean ± SD)
1	–	7	59.47 ± 0.17b
2	51.46 ± 0.26c	8	29.46 ± 0.67g
3	39.46 ± 0.31d	9	26.800 ± 0.00h
4	32.29 ± 0.24f	10	31.560 ± 0.41f
5	65.74 ± 0.18a	11	18.960 ± 1.31j
6	34.72 ± 0.28e	12	24.250 ± 0.69i

− The fraction had no inhibitory effect on pathogens. Different lowercase letters in the figure indicate that the *p* < 0.05 level was significantly different.

### 3.2 Structural identification

**Figure 2:**
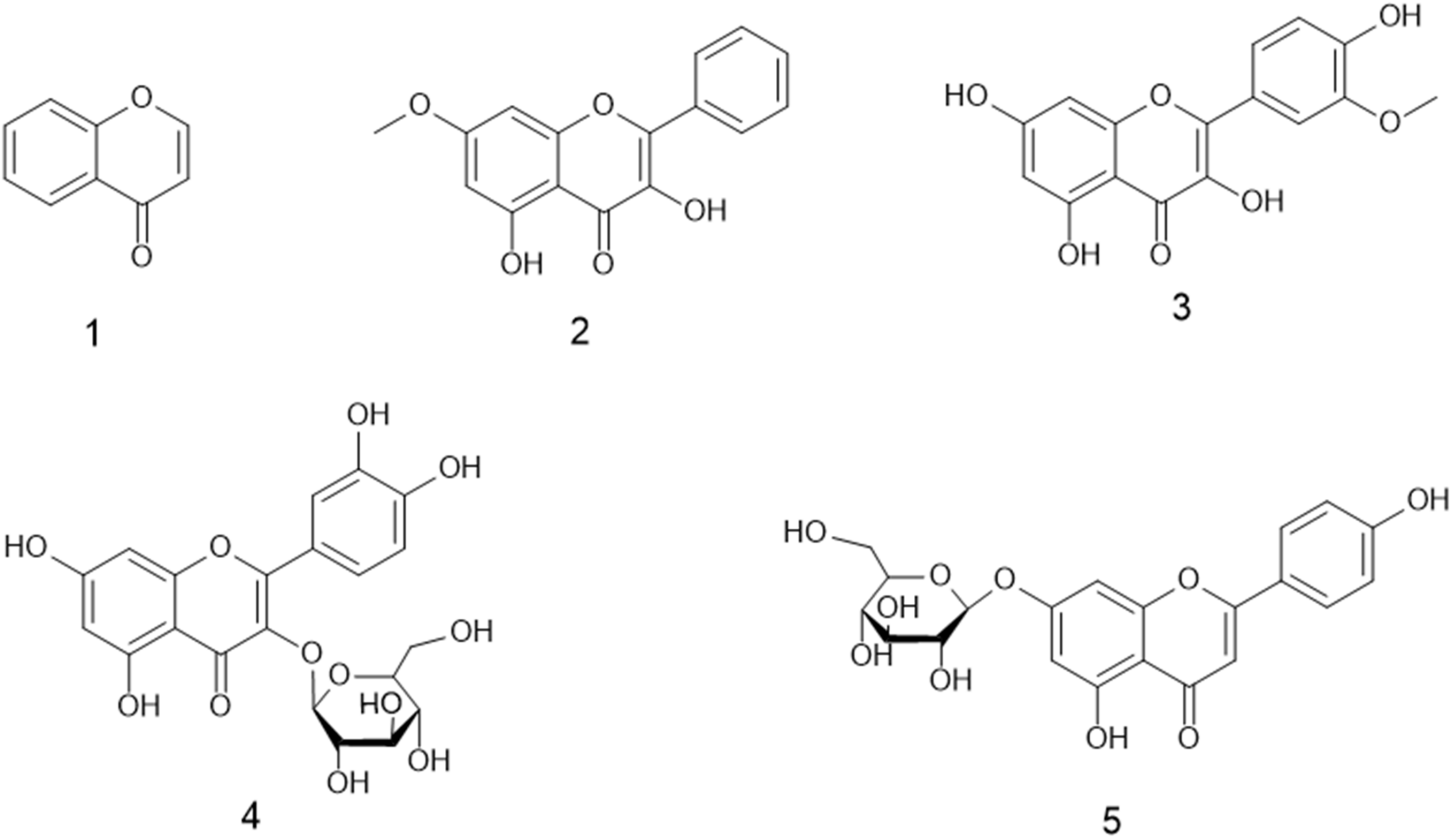
Structures of compounds isolated and identified from *Humulus scandens.* Note: Chromone (1), tectochrysin (2), isorhamnetin (3), hyperoside (4), and apigenin 7-glucoside (5).

The structure of the compounds is shown in **Figure 2**.

Compound 1—chromone, white amorphous powder: The ^13^C-NMR and DEPT of the compound showed that it had nine carbon signals, of which six were methines and three were quaternary carbons. The molecular formula was determined as C_9_H_6_O_2_ by ESI-MS. The nuclear magnetic spectrum data are as follows: ^1^H-NMR (500 MHz, DMSO) δ: 8.28 (dd, J = 6.0, 0.6 Hz, 1H), 8.02 (dd, J = 8.0, 1.7 Hz, 1H), 7.78 (m, 1H), 7.62 (dd, J = 8.5, 0.9 Hz, 1H), 7.46 (tt, J = 7.8, 0.8 Hz, 1H), 6.33 (d, J = 6.2 Hz, 1H), ^13^C-NMR (500 MHz, DMSO) δ: 157.01 (C-1), 118.52 (C-2), 134.28 (C-3), 125.52 (C-4), 124.96 (C-5), 124.27 (C-6), 176.40 (C-7), 112.30 (C-8), 156.01 (C-9). The data were basically consistent with the reference (Morimoto et al., 2003), and the structure of compound 1 was determined.

Compound 2—tectochrysin, yellow non-crystalline powder: The ^13^C-NMR and DEPT of the compound showed that it had 16 carbon signals, which were 1 methyl, 8 methines, and 7 quaternary carbons, respectively. The molecular formula was determined to be C_16_H_12_O_4_ by ESI-MS. The nuclear magnetic spectrum data are as follows: ^1^H-NMR (500 MHz, DMSO) δ: 8.08 (m, 2H), 7.60 (m, 3H), 7.02 (d, J = 1.6 Hz, 1H), 6.80 (t, J = 2.1 Hz, 1H), 6.38 (d, J = 2.3 Hz, 1H), 3.86 (d, J = 1.4 Hz, 3H), 3.31 (s, 4H), ^13^C-NMR (500 MHz, DMSO) δ: 56.15 (C-OMe), 163.51 (C-2), 105.39 (C-3), 182.14 (C-4), 161.21 (C-5), 98.20 (C-6), 165.41 (C-7), 92.87 (C-8), 157.42 (C-9), 104.97 (C-10), 130.63 (C-1′), 126.50 (C-2′,6′), 129.18 (C-3′,5’), 132.20 (C-4′). The data were basically consistent with the reference (Xu et al., 2009), and the structure of compound 2 was determined.

Compound 3—isorhamnetin, yellow non-crystalline powder: The ^13^C-NMR and DEPT of the compound showed that it had 16 carbon signals, which were 1 methyl, 8 methines, and 7 quaternary carbons, respectively. The molecular formula was determined to be C_16_H_12_O_4_ by ESI-MS. The nuclear magnetic spectrum data are as follows: ^1^H-NMR (500 MHz, DMSO) δ: 12.44 (s, 1H), 7.74 (d, J = 2.1 Hz, 1H), 7.67 (dd, J = 8.5, 2.1 Hz, 1H), 6.92 (d, J = 8.5 Hz, 1H), 6.46 (d, J = 2.1 Hz, 1H), 6.17 (d, J = 2.4 Hz, 1H), 3.83 (s, 3H), ^13^C-NMR (500 MHz, DMSO) δ: 55.77 (C-OMe), 146.59 (C-2), 135.82 (C-3), 175.87 (C-4), 160.68 (C-5), 98.27 (C-6), 164.12 (C-7), 93.64 (C-8), 156.19 (C-9), 102.96 (C-10), 121.99 (C-1′), 111.68 (C-2′), 147.38 (C-3′), 148.81 (C-4′), 115.55 (C-5′), 121.71 (C-6′). The data were basically consistent with the reference (Dayem et al., 2015), and the structure of compound 3 was determined.

Compound 4—hyperoside, yellow non-crystalline powder: The ^13^C-NMR and DEPT of the compound showed that it had 21 carbon signals, which were 1 methylene, 10 methines, and 10 quaternary carbons, respectively. The molecular formula was determined to be C_21_H_20_O_12_ by ESI-MS. The nuclear magnetic spectrum data are as follows: ^1^H-NMR (500 MHz, DMSO) δ: 12.61 (s, 2H), 7.65 (dd, J = 8.4, 2.3 Hz, 2H), 7.50 (d, J = 2.2 Hz, 2H), 6.79 (d, J = 8.5 Hz, 2H), 6.38 (d, J = 2.0 Hz, 2H), 6.17 (d, J = 2.0 Hz, 2H), 5.35 (d, J = 7.7 Hz, 2H), 5.10 (s, 1H), 4.83 (s, 1H), 4.41 (s, 2H), 3.62 (d, J = 3.4 Hz, 2H), 3.54 (t, J = 8.7 Hz, 2H), 3.42 (dt, J = 13.1, 6.6 Hz, 3H), 3.35 (d, J = 3.3 Hz, 4H), 3.27 (dd, J = 16.0, 5.8 Hz, 26H), 3.14 (s, 1H), ^13^C-NMR (500 MHz, DMSO) δ: 177.49 (C-4), 164.19 (C-7), 161.24 (C-5), 156.31 (C-9), 156.23 (C-2), 148.49 (C-4′), 144.85 (C-3′), 133.48 (C-3), 122.02 (C-6′), 121.10 (C-1′), 115.93 (C-5′), 115.19 (C-2′), 103.91 (C-10), 101.79 (C-1Gal), 98.69 (C-6), 93.51 (C-8), 75.86 (C-5Gal), 73.19 (C-3Gal), 71.21 (C-2Gal), 67.93 (C-4Gal), 60.15 (C-6Gal). The data were basically consistent with the reference (Ma et al., 2007), and the structure of compound 4 was determined.

Compound 5—apigenin 7-glucoside: The ^13^C-NMR and DEPT of the compound showed that it had 21 carbon signals, which were 1 methylene, 10 methylenes, and 10 quaternary carbons, respectively. The molecular formula was determined to be C_21_H_20_O_10_ by ESI-MS. The nuclear magnetic spectrum data are as follows: ^1^H-NMR (500 MHz, DMSO) δ: 12.95 (d, J = 1.2 Hz, 1H), 10.39 (s, 1H), 7.95 (m, 2H), 6.93 (m, 2H), 6.86 (d, J = 1.1 Hz, 1H), 6.82 (m, 1H), 6.43 (m, 1H), 5.39 (dd, J = 5.0, 1.1 Hz, 1H), 5.12 (dd, J = 4.9, 1.1 Hz, 1H), 5.06 (m, 2H), 4.60 (t, J = 5.6 Hz, 1H), 3.70 (dd, J = 10.9, 5.2 Hz, 1H), 3.45 (ddd, J = 14.6, 9.3, 5.8 Hz, 2H), 3.22 (m, 10H), 2.53 (d, J = 1.2 Hz, 1H), ^13^C-NMR (500 MHz, DMSO) δ: 182.04 (C-4), 164.27 (C-2), 162.98 (C-7), 161.40 (C-5), 161.13 (C-4′), 156.96 (C-9), 128.65 (C-2′,6′), 121.04 (C-1′), 116.02 (C-3′,5′), 105.35 (C-10), 103.13 (C-3), 99.90 (C-1Gal), 99.53 (C-6), 94.86 (C-8), 77.20 (C-3Gal), 76.46 (C-5Gal), 73.11 (C-2Gal), 69.55 (C-4Gal), 60.61 (C-6Gal). The data were basically consistent with the reference (Ma et al., 2009), and the structure of compound 5 was determined.

### 3.3 Antimicrobial activity of the compounds

**Figure 3:**
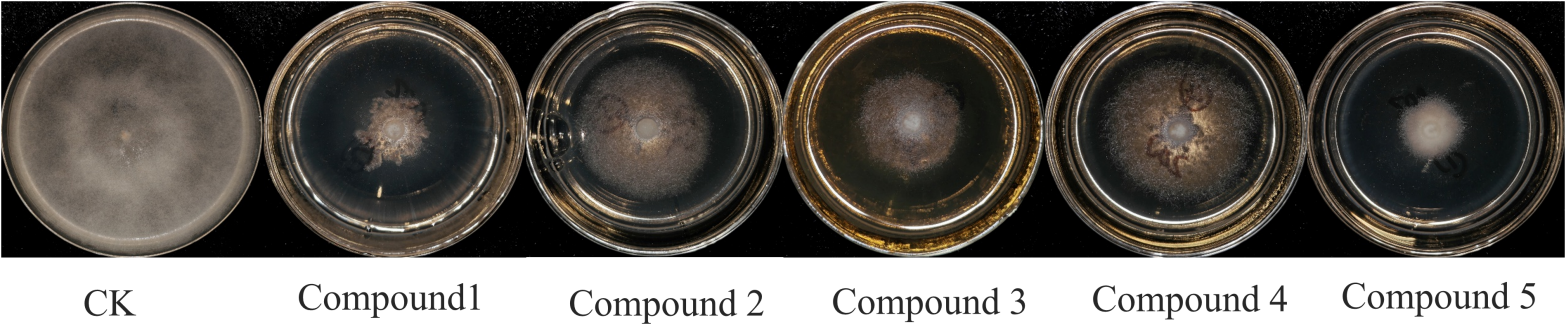
Screening of the antimicrobial activity of compounds against *Phytophthora nicotianae.* The treatment concentration was 100 μg/mL.

The antimicrobial activity of the five isolated compounds is shown in **Figure 3** and **Table 3**. At the concentration of 100 μg/mL, compounds 1, 3, and 5 had better inhibitory effects, and the inhibition rates were 81.11%, 60.00%, and 83.33%, respectively. The inhibitory effect of compounds 2 and 4 was not obvious, and the inhibition rate was between 34% and 35%.

**Table 3:** Inhibition rate of compounds against *Phytophthora nicotianae*.

Phytopathogenic fungi	Inhibition rate (%; mean ± SD)				
	Compound 1	Compound 2	Compound 3	Compound 4	Compound 5
*P. nicotianae*	81.11 ± 0.41b	33.33 ± 0.52e	60.00 ± 0.77c	35.00 ± 1.25d	83.33 ± 0.40a

Different lowercase letters in the figure indicate that the *p* < 0.05 level was significantly different.

**Figure 4:**
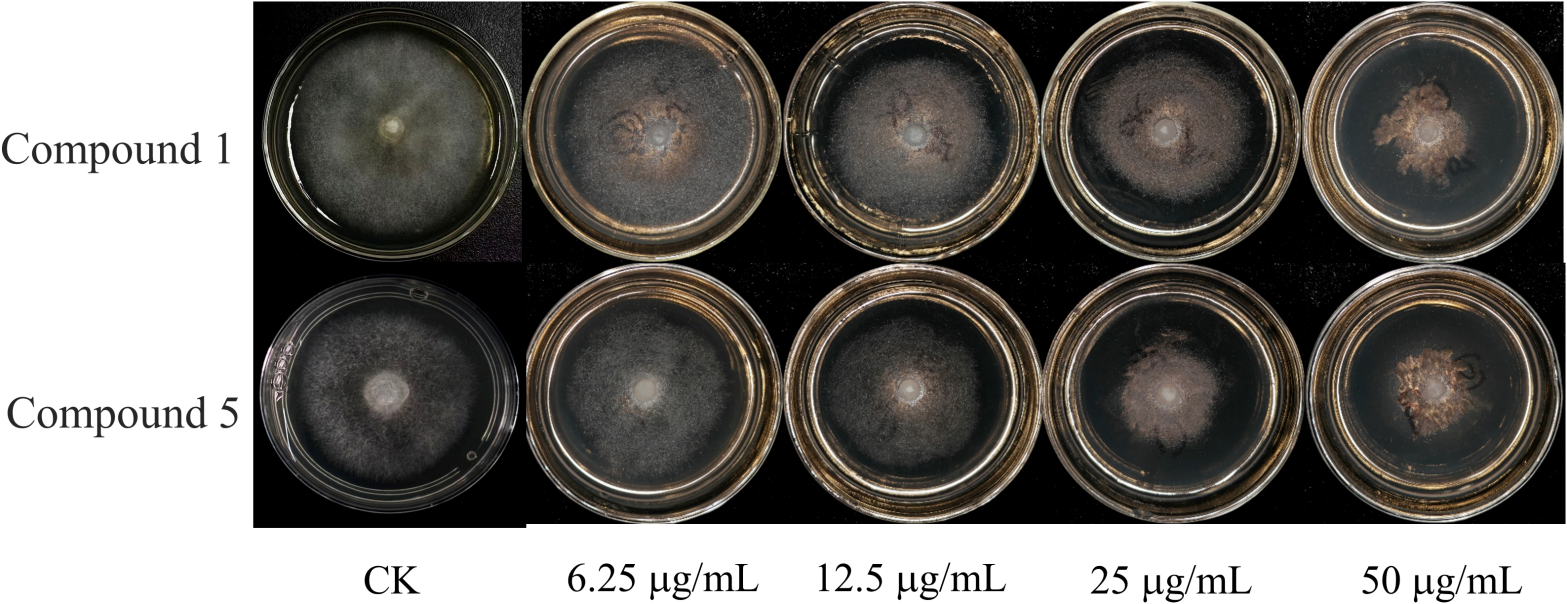
Inhibitory activities of compounds 1 and 5 on the mycelial growth of *Phytophthora nicotianae*.

Therefore, compounds 1 and 5 were selected for further virulence determination of *P. nicotianae*. The results are shown in **Figure 4** and **Table 4**. There was a significant concentration relationship between the inhibitory effect of the compounds on *P. nicotianae*. The higher the concentration of the compounds, the stronger the antimicrobial activity. The EC_50_ values of compounds 1 and 5 were 51.70 and 31.71 μg/mL, respectively. Compound 5 exhibits a lower EC_50_, indicative of higher inhibitory activity.

**Table 4:** Toxicity determination of compounds 1 and 5 against *Phytophthora nicotianae*.

Compounds	Virulence equation	EC_50_ (μg/mL)	Correlation coefficient (*R*^2^)
1	*Y* = 0.8239*X* + 7.408	51.70	0.9048
5	*Y* = 0.5370*X* + 32.97	31.71	0.9115

The compound was repeated three times against the pathogen.

### 3.4 Determination of MIC

The minimum inhibitory concentration (MIC) of the compounds is shown in **Table 5**. When the concentration of compound 1 was 0~200 μg/mL, the mycelium growth could still be seen, while the mycelium growth could not be seen at 400 μg/mL; when the concentration of compound 5 was 0~100 μg/mL, the mycelium growth was still visible, while the mycelium growth was not seen at 200 μg/mL. The MICs of compounds 1 and 5 against *P. nicotianae* were 400 and 200 μg/mL, respectively.

**Table 5:** The MIC of compounds 1 and 5 against *Phytophthora nicotianae*.

Compounds	Concentration (μg/mL)							
	0	12.5	25	50	100	200	400	800
1	+++	+++	+++	++	++	+	−	−
5	+++	+++	++	++	++	−	−	−

“+++”: a large amount of mycelium growth; “++”: a moderate amount of mycelium growth; “+”: a small amount of mycelium growth; “−”: no mycelium growth.

### 3.5 Effects of compounds on mycelial morphology of *Phytophthora nicotianae*

The EC_50_ of compounds 1 and 5 was selected as the experimental concentration to observe the effect of compounds on mycelial morphology.

**Figure 5:**
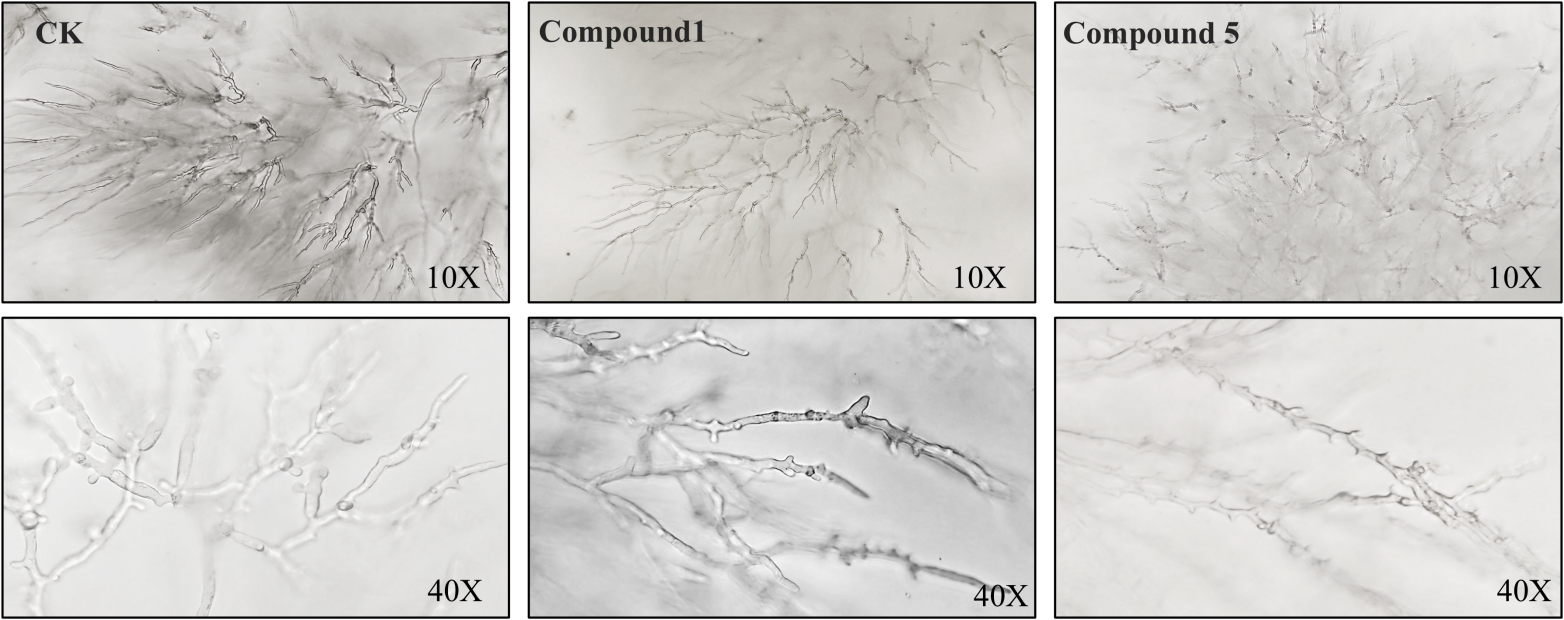
Effects of compounds 1 and 5 on mycelial morphology of *P. nicotianae* observed via an electron microscope.

The results of optical electron microscopy are shown in **Figure 5**. The surface of CK-treated mycelia was smooth, uniform in thickness, and maintained a good growth state. The surface of the mycelium treated with the compound was rough, the thickness of the mycelium was uneven, the binary branch was significantly shortened, and the growth was deformed.

**Figure 6:**
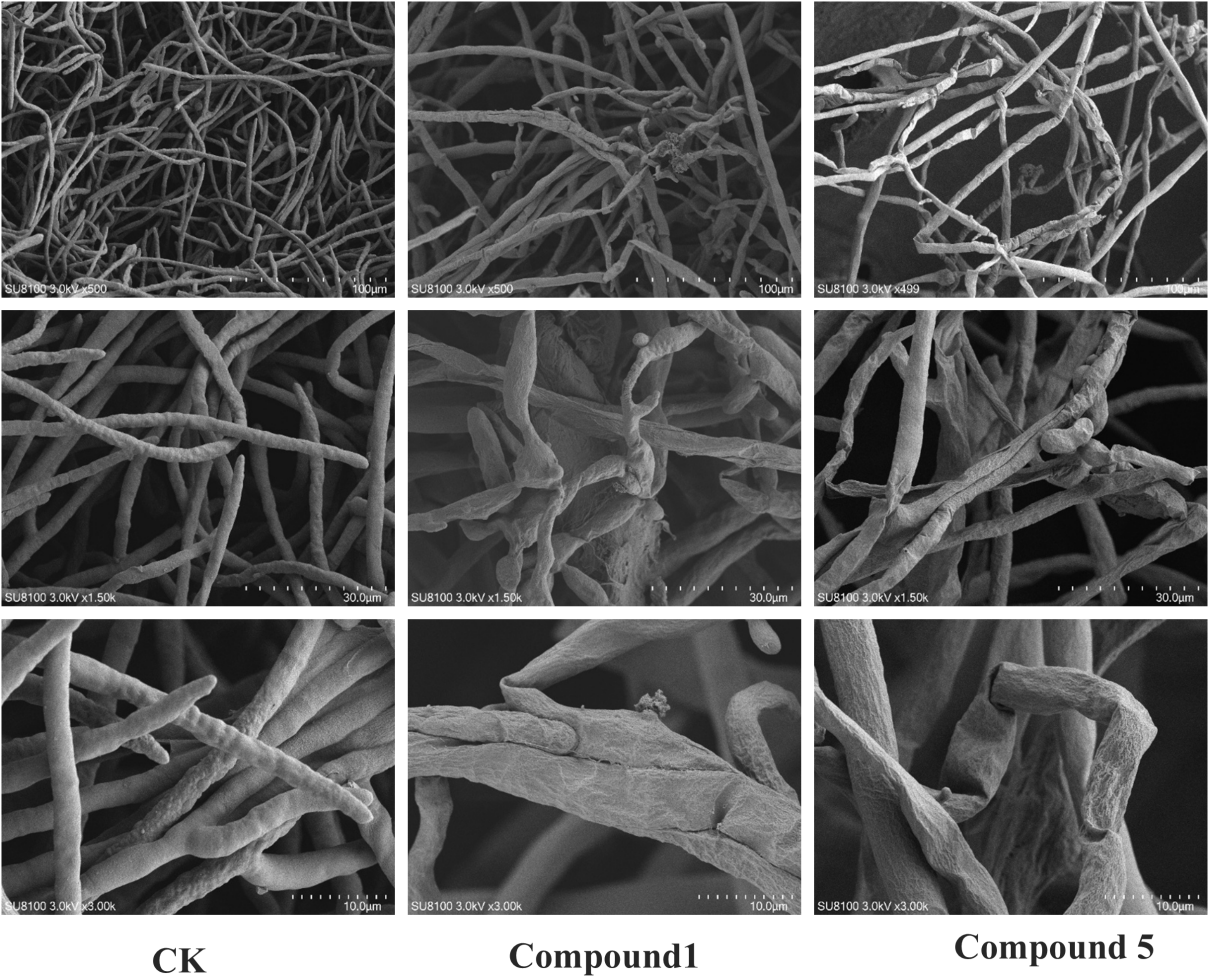
Scanning electron micrographs of *Phytophthora nicotianae* treated with compounds at various fractions.

The results of scanning electron microscopy are shown in **Figure 6**. The mycelium of the control group was smooth, full, and uniform in thickness, while the surface of the mycelium treated with the compound was rough, dehydrated and shrunk, uneven in thickness, irregularly distorted, and the overall morphology changed.

### 3.6 Effect of compounds on the cell membrane permeability of *Phytophthora nicotianae*

**Figure 7:**
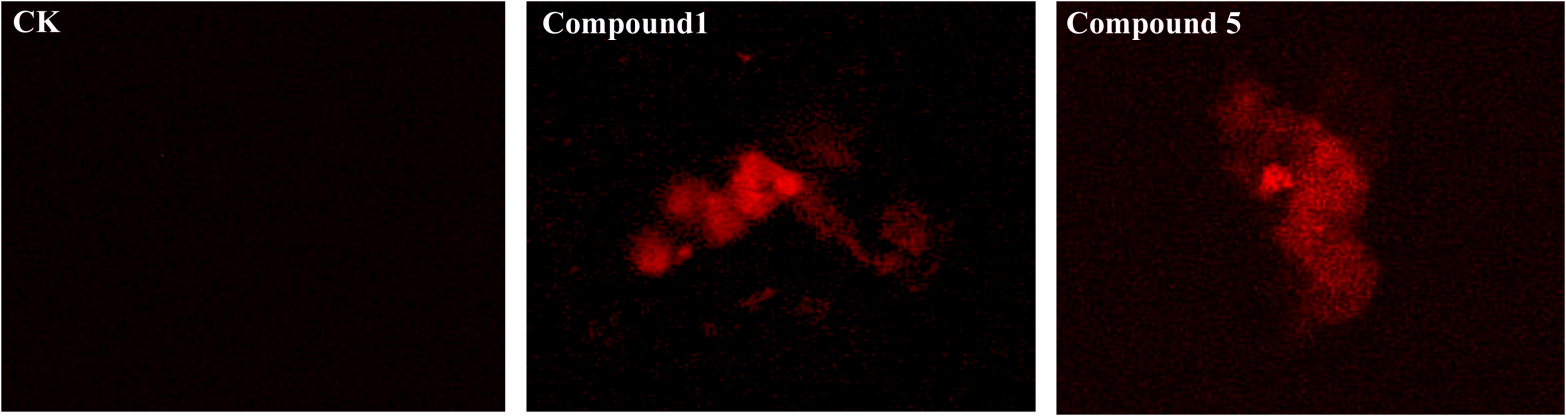
Effects of compounds on cell membrane permeability of *Phytophthora nicotianae*.

The cell membrane permeability was detected, and the results are shown in **Figure 7**. Under dark-field fluorescence excitation conditions, no fluorescent signal was detected in the mycelia of the control group, indicating that the cell membrane structure is intact. The mycelium treated with the compound showed significant red fluorescence, indicating that the cell membrane was damaged, resulting in increased membrane permeability, and PI entered the cell through the damaged cell membrane.

## 4 Discussion and conclusions

Previous investigations have established *H. scandens*’ insecticidal and herbicidal properties (Gao et al., 2008, 2009; Tao et al., 2011). However, its potential against phytopathogenic microorganisms remains comparatively unexplored. This study identifies and characterizes specific antimicrobial constituents from the *H. scandens* EtOAc fraction, laying a foundation for developing botanical antimicrobials against *P. nicotianae*. While the EtOAc crude extract demonstrated broad-spectrum inhibition against 10 pathogens—notably against *P. nicotianae* (82.81% inhibition rate)—the relationship between isolated monomers and observed bioactivity reveals complexities requiring nuanced interpretation.

The comparable efficacy between the EtOAc crude extract (82.81% inhibition) and isolated compounds (e.g., compound 5: 83.33%; compound 1: 81.11%) suggests that monomeric constituents may not exclusively account for the bioactivity. Although synergistic interactions among phytochemicals could enhance collective potency (Kukhtenko et al., 2024), alternative explanations warrant consideration, e.g., concentration disparities (the crude extract was tested at 3 mg/mL, while compounds were evaluated at 100 μg/mL, complicating direct potency comparisons) and extraction artifacts (polar solvents like methanol may alter compound stability or conformation during isolation). In addition, minor constituents such as untracked components (terpenoids or alkaloids) discarded during fractionation may contribute to activity (Wang et al., 2024). While these synergistic effects remain plausible, methodological limitations prevent definitive conclusions. Future studies should employ isobolographic analyses to empirically validate interactions among isolated fractions.

The functional group is the key to determining the properties of the compound, and the binding of hydroxyl (Yuan et al., 2024), methoxy (Zhang and Go, 2011), and glucose (Liu Y. et al., 2011) at different sites will affect its activity. This study found that the crude extract of the EtOAc layer showed certain activity against 10 pathogens, among which the inhibitory effect on *P. nicotianae* was better (82.81%). Therefore, the activity tracking separation of the EtOAc layer extract was carried out, and five compounds were obtained, all of which were flavonoids. Earlier reports have shown that flavonoids have good antimicrobial activity against *P. nicotianae* (Sun et al., 2022; Li et al., 2021; Su et al., 2023). The EC_50_ of compound 1 (51.70 μg/mL) was higher than that of compound 5 (31.71 μg/mL), indicating that compound 5 had stronger antimicrobial activity. It is speculated that the difference in activity is due to the difference in chemical structure. Compound 1 has no hydroxyl group and no sugar group, while compound 5 contains multiple hydroxyl groups and glucose groups. Polyhydroxy can increase molecular polarity, increase hydrogen bond binding sites, and improve antimicrobial activity (Faria et al., 2012). The glycosyl group enhances the interaction between the compound and the glycoside on the surface of *P. nicotianae*, thereby forming a stable target to increase activity (Quave et al., 2023). In addition, comparing the results of this study with other studies can also show that there is a correlation between functional groups and the activity of compounds. The EC_50_ of compound 5 (31.71 μg/mL) was higher than that of naringenin (20.19 μg/mL) (Li et al., 2021), indicating that the antimicrobial activity of naringenin was higher than that of compound 5. From the analysis of its structure, naringenin is a dihydroflavonoid glycoside with a saturated C ring, high fat solubility, stronger permeability to the membrane, and better antimicrobial activity. Compound 5 is a flavonoid glycoside, which has lower activity than naringenin (Yan et al., 2024).

At present, the exploration of the antimicrobial mechanism of flavonoids mainly focuses on the integrity of the cell membrane, energy metabolism, and protein synthesis (Da Costa and Do Nascimento, 2025). Flavonoids can affect the expression of genes related to the biosynthesis of the cell wall and cell membrane of pathogenic bacteria or directly destroy the cell wall and cell membrane, resulting in the loss of intracellular substances and the inhibition of energy metabolism processes such as respiratory chain and ATP synthesis, eventually leading to bacterial death (Zhou et al., 2022; Yi et al., 2021; Castano-Duque et al., 2024). Here, we found that the surface characteristics and integrity of the mycelium treated with the compound were damaged to a certain extent, and the cell membrane of the pathogen was destroyed by electron microscopy and PI fluorescence coloration. This is consistent with the results of previous studies (Gu and Liang, 2023), but it is still necessary to further verify whether compound 5 will interfere with the metabolism and energy metabolism of bacteria.

This study acknowledges constraints; exclusive *in vitro* validation limits agricultural applicability; confirmed membrane disruption lacks verification of energy/proteomic impacts; and potential synergies remain untested. Despite this, apigenin 7-glucoside (5) emerges as a potent anti-oomycete lead (MIC = 200 μg/mL). Future work should prioritize formulation optimization for field deployment, transcriptomic target identification, and integrated management trials combining compound 5 with biocontrol strategies for tobacco black shank.

This work establishes *H. scandens* as a source of anti-*Phytophthora* flavonoids, with apigenin 7-glucoside representing a viable template for botanical fungicide development. By contextualizing findings within structural and mechanistic frameworks—while acknowledging methodological and biological complexities—we provide a balanced foundation for sustainable disease management strategies.

## Data Availability

The original contributions presented in the study are included in the article/**Supplementary Material**. Further inquiries can be directed to the corresponding authors.
