# Supplementary material for: Isolation, identification, and antimicrobial effect analysis of active components from Humulus scandens against Phytophthora nicotianae
Source: Front Plant Sci. 2026 Mar 5;17:1753587. doi: 10.3389/fpls.2026.1753587 (PMC12999855; doi:10.3389/fpls.2026.1753587)

Supplementary Material

Figure S1 ^1^H NMR spectrum of compound 1 in DMSO

Figure S2 ^13^C NMR spectrum of compound 1 in DMSO

Figure S3 ^1^H NMR spectrum of compound 2 in DMSO

Figure S4 ^13^C NMR spectrum of compound 2 DMSO

Figure S5 ^1^H NMR spectrum of compound 3 in DMSO

Figure S6 ^13^C NMR spectrum of compound 3 in DMSO

Figure S7 ^1^H NMR spectrum of compound 4 in DMSO

Figure S8 ^13^C NMR spectrum of compound 4 in DMSO

Figure S9 ^1^H NMR spectrum of compound 5 in DMSO


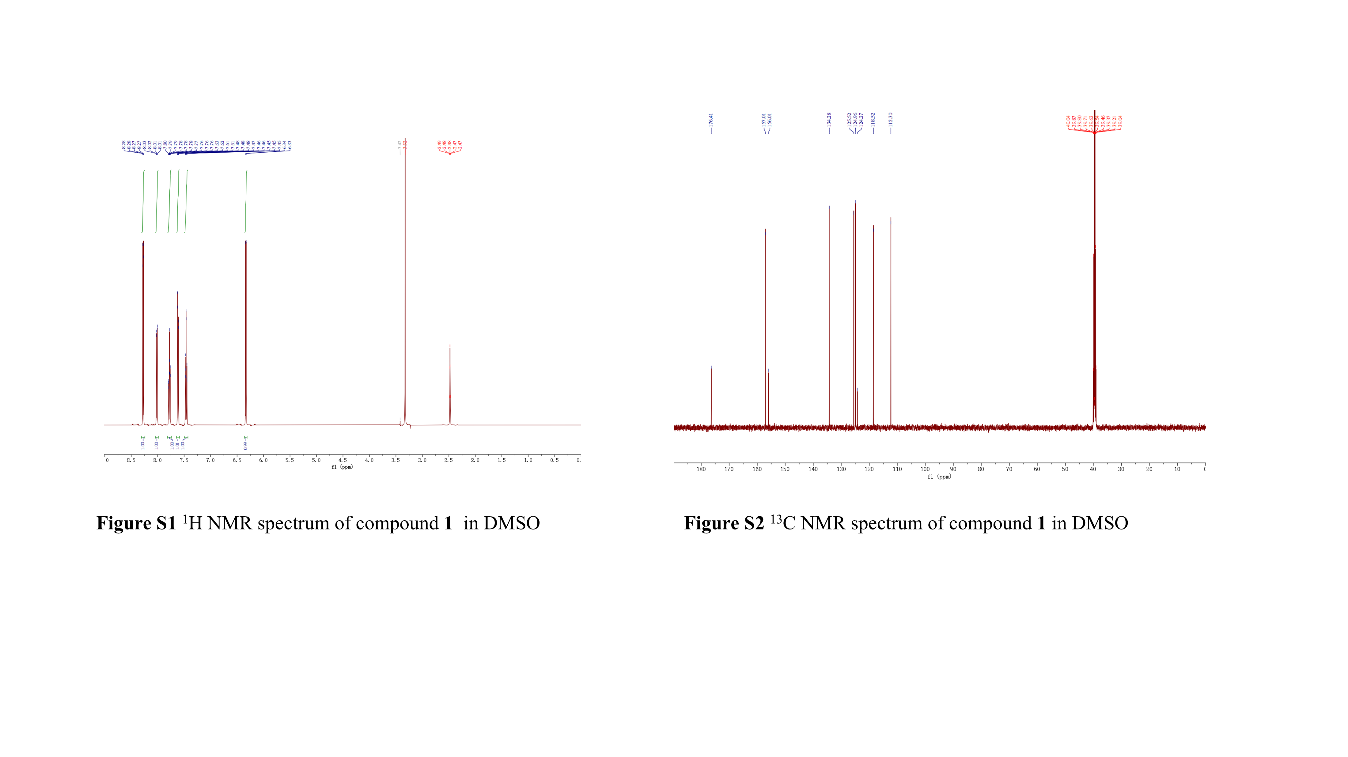
Figure S10 ^13^C NMR spectrum of compound 5 in DMSO


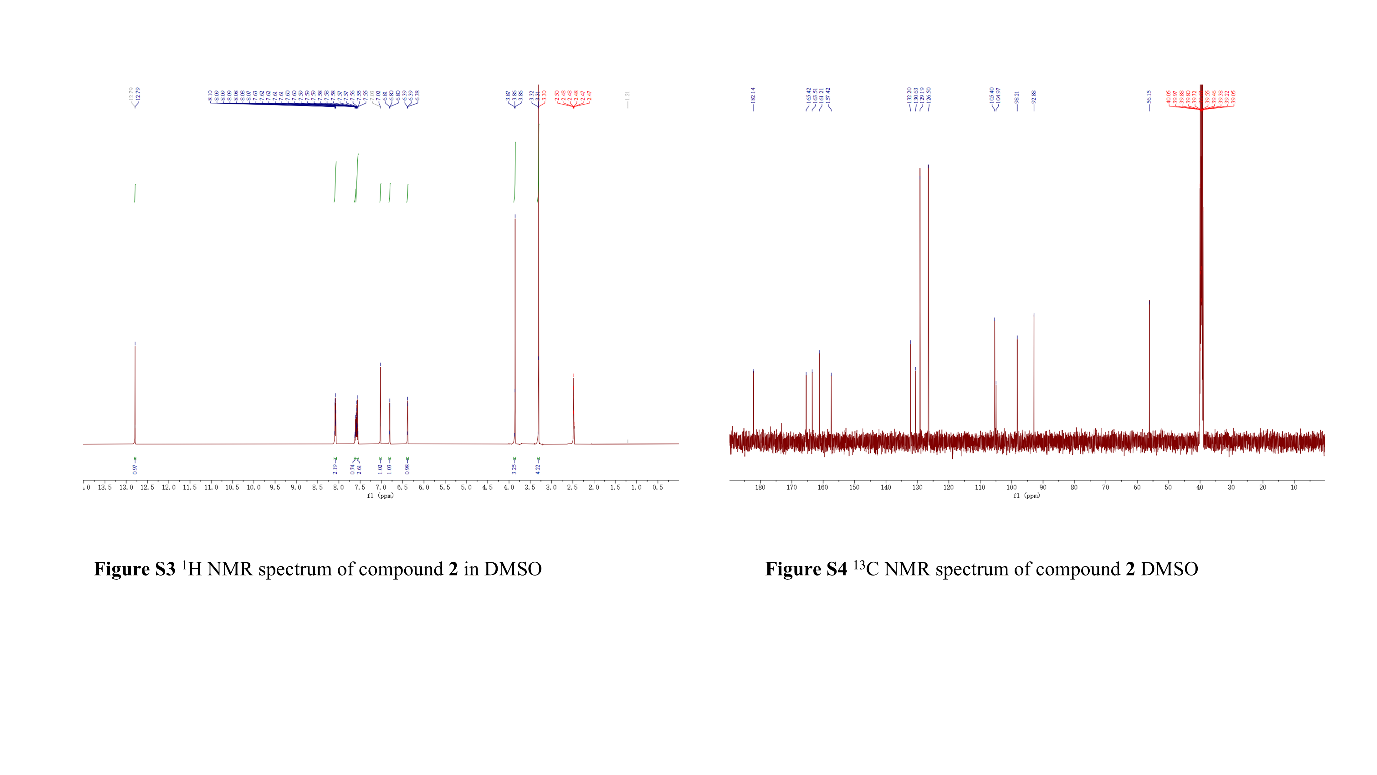


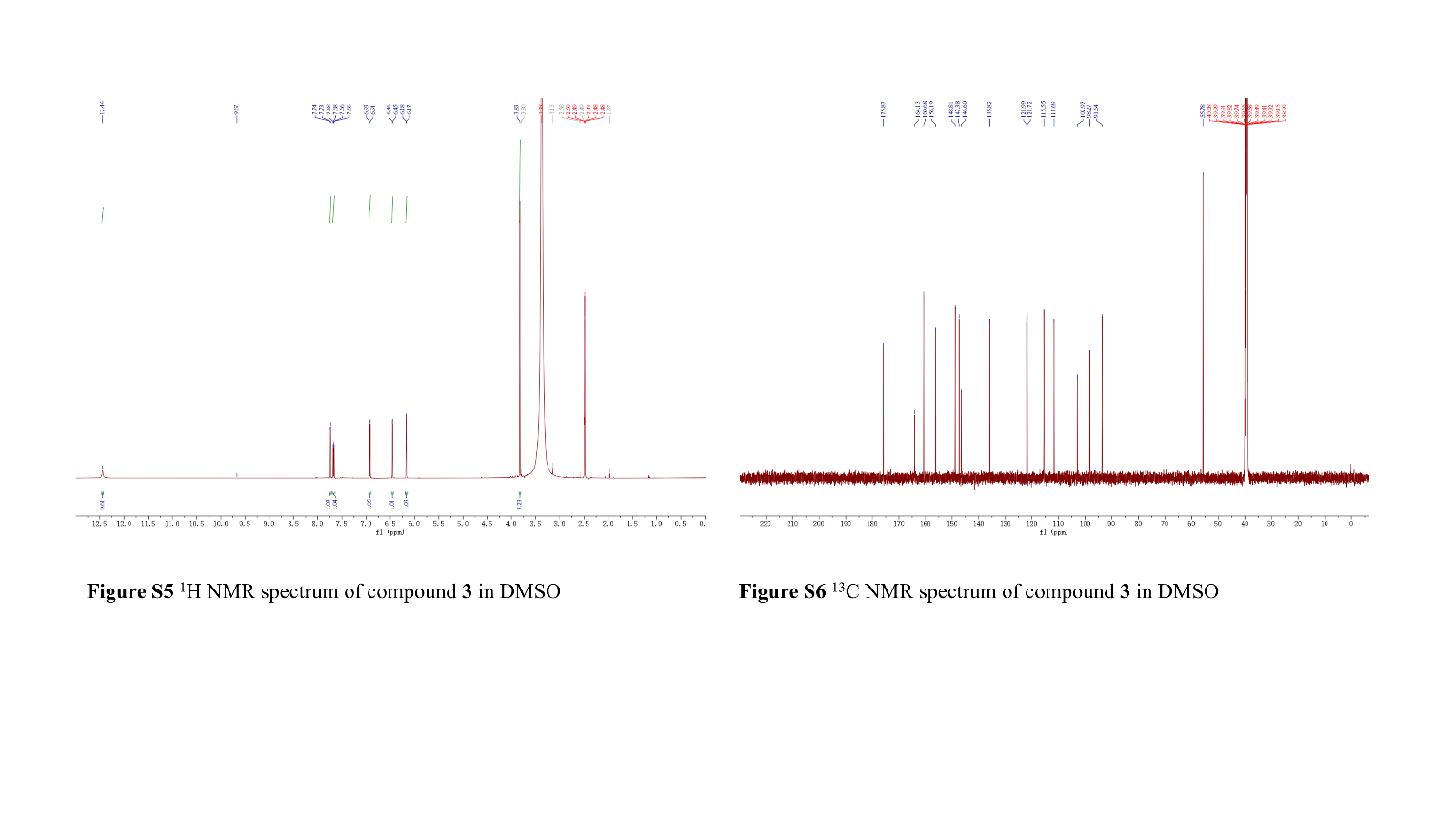


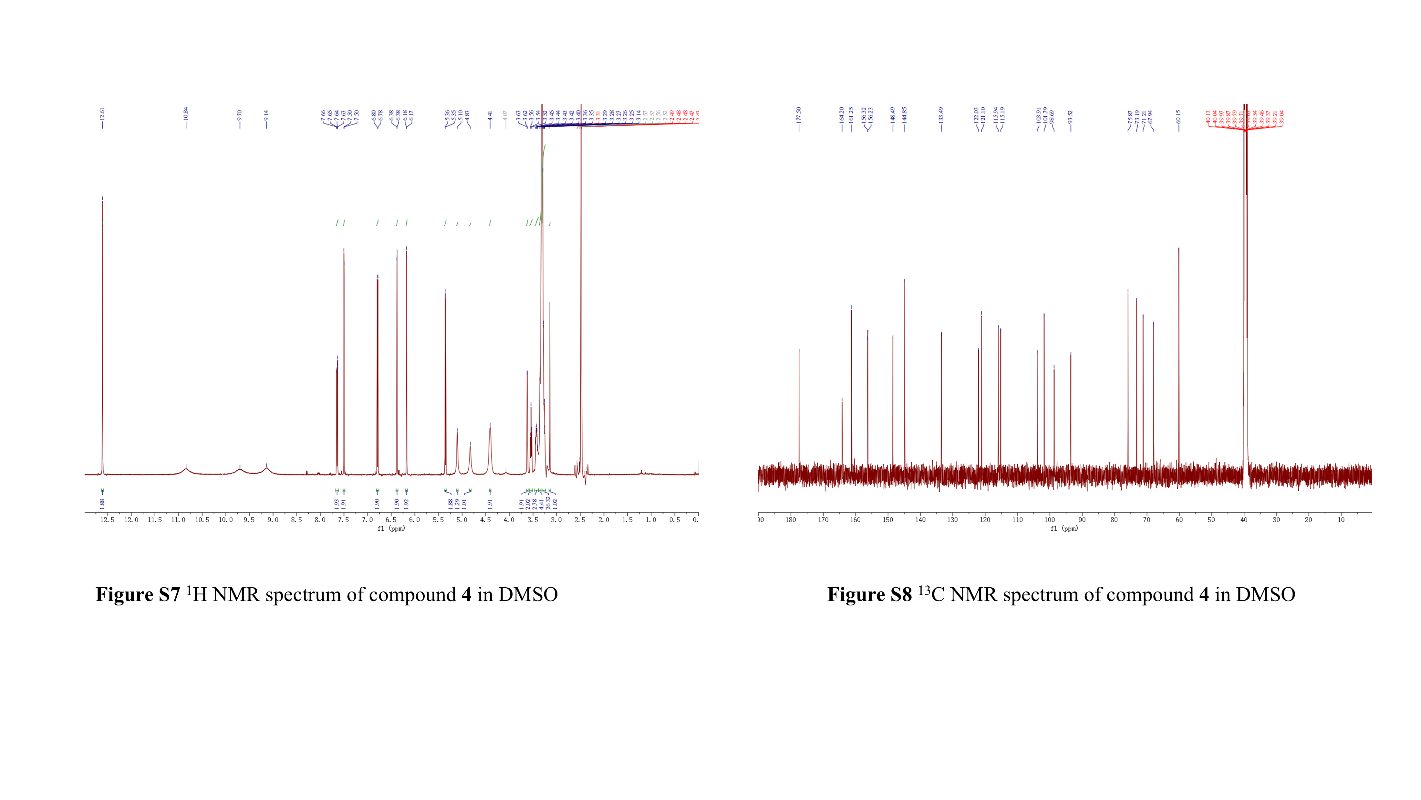


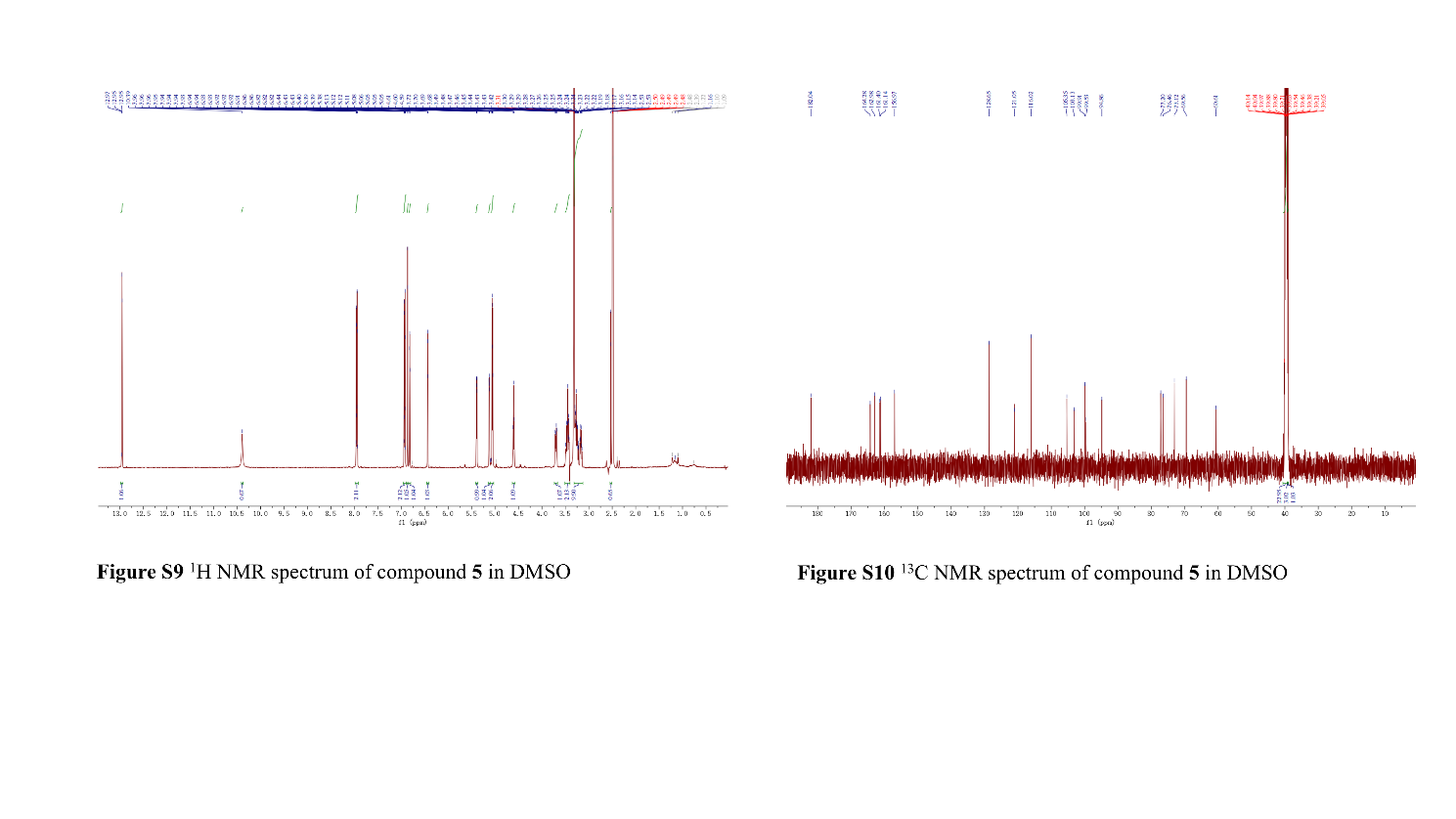

Supplement: Supplementary file 1 [file DataSheet1.docx]
